# Trichotillomania and Dermatitis Artefacta: A Rare Coexistence

**DOI:** 10.1155/2012/674136

**Published:** 2012-02-09

**Authors:** Neeraj Varyani, Sunny Garg, Garima Gupta, Shivendra Singh, Kamlakar Tripathi

**Affiliations:** ^1^Department of General Medicine, Institute of Medical Sciences, Banaras Hindu University, Varanasi 221005, India; ^2^Department of Obstetrics and Gynaecology, Institute of Medical Sciences, Banaras Hindu University, Varanasi 221005, India; ^3^Department of Nephrology, Institute of Medical Sciences, Banaras Hindu University, Varanasi, Uttar Pradesh 221005, India

## Abstract

A 24-year-old pregnant female patient presented with complains of bilateral lower limb swelling and fever for 1 month. On examination, blood pressure was 144/94 mmHg along with pitting pedal edema. She had bizarre skin lesions, aligned longitudinally and distributed over the approachable site of the body with tapering ends and in various stages of healing. Lower limbs examination also revealed similar lesions with signs of cellulitis. Her scalp had short and distorted hair suggesting pulling and plucking. These skin lesions and the denial of self-infliction by the patient made us reach the diagnosis of dermatitis artefacta with trichotillomania. Psychotherapy was advocated along with conservative management of skin lesions. The patient improved and is under our follow up.

## 1. Introduction

Psychocutaneous disorders include a vast spectrum of dermatological diseases that are somatic manifestations of the patient disordered thinking, behaviour, or perception. Although the relationship between dermatological disorders and the psychological status of the patient has been known since ages, large studies on treatment of these disorders are lacking. Our case highlights the importance of a strong suspicion for an underlying psychiatric illness when a clinical presentation could not be explained by any known medical condition.

## 2. Case Report

In September 2011, a 24-year-old eight-month primigravida, with last menstrual period on 24th January, 2011, presented to our outpatient department with complaints of swelling of both lower limbs and fever for the past one month. The swelling was symmetrical on onset, progressive, with associated pain and redness of overlying skin. She denied history of headache, palpitations, breathlessness, cough, orthopnea, paroxysmal nocturnal dyspnea, jaundice, decreased urine output, or dysuria. There was no clinically significant menstrual and obstetrical history. She denied smoking or alcohol consumption and illicit drug abuse. While she narrated her complaints, we noticed a sense of hesitancy in her speech and difficulty in making an eye contact.

**Figure 1:**
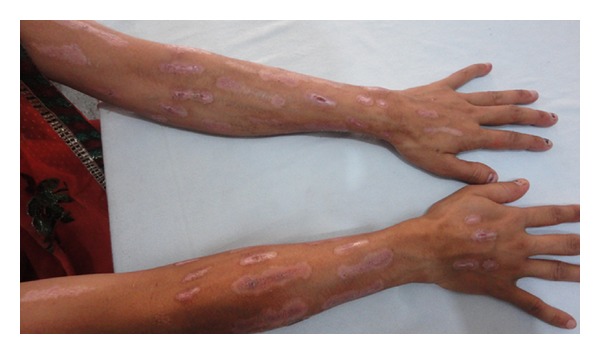
Dermatitis artefacta lesions on the upper limbs.

**Figure 2:**
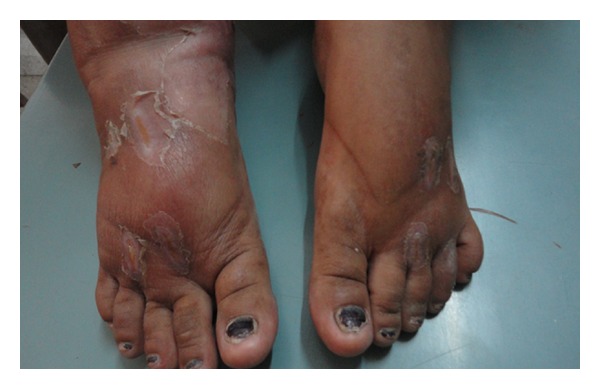
Dermatitis artefacta lesions on the bilateral feet.

**Figure 3:**
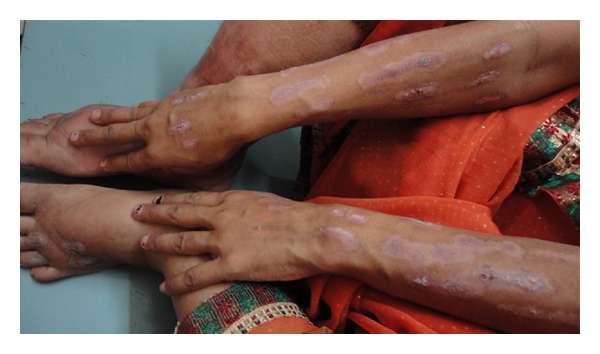
Confluent dermatitis artefacta lesions on the upper and lower limbs.

**Figure 4:**
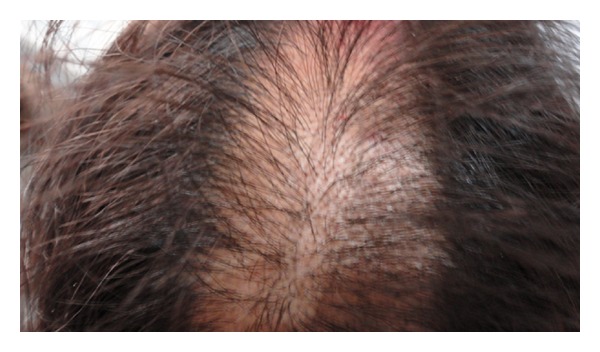
Trichotillomania lesions on vertex of scalp.

On examination, the patient was conscious, cooperative and well oriented. She was hypertensive (blood pressure 144/94 mm of Hg) on two separate occasions. She was febrile (102 degrees Fahrenheit) with sinus tachycardia. Her fundus examination was normal and there was no thyroid swelling. Objective signs of inflammation like swelling, redness, and tenderness of bilateral feet were present, suggesting cellulitis. We noticed multiple, well-demarcated, bizarre lesions on the dorsum of the bilateral forearms (Figures 1 and 3) and aligned longitudinally. The lesions had tapering ends and were in various stages of healing and could not be explained by any known dermatological condition. This made us suspicious for an afflicted physical violence on her. On detailed dermatological survey, these lesions were located on the easily approachable aspect of bilateral upper limbs and dorsum of bilateral feet (Figures 2 and 3), with sparing of the usually covered and unapproachable areas of limbs, trunk, and genitalia. Also, there was an area of short, irregular, broken, and distorted hair on the scalp, mainly over the vertex, suggesting hair pulling and plucking (Figure 4), whereas eyebrows, eyelashes, pubic hair, and other body hair were spared. This characteristic distribution of the lesions displayed signs of self-inflicted wounds rather than physical violence.

 On further inquest, although she admitted the appearance of these lesions in episodes since the past four years, surprisingly she denied any knowledge of the origin, cause, or circumstances in which these skin lesions appeared or progressed, neither did she admit their self-infliction. There was no history of any psychiatric illness or similar behavioural disorder in her family. She also denied history of antipsychotic drug intake. Detailed history from her family members did not reveal any evidence of delayed developmental milestones, poor scholastic performance, child abuse, marital dispute, or loss of any close relative in the recent past. Personal counselling revealed loss of interest in routine household activities and voluntary plucking of her hair with a sense of tension and anxiety before the act followed by a sense of pleasure, relief, and gratification. But even then she did not admit the self-infliction of the lesions on the skin.

Hematological parameters revealed anemia (hemoglobin 85 g/L), leucocytosis (15 × 10^9^/L), and hypoalbuminemia (18 g/L). The rest of haematological profile was normal. Urinalysis revealed proteinuria (0.8 g/d). Sonography of the abdomen showed a live intrauterine gestation of 28 + 4 weeks without any evidence of trichobezoars. Based on the clinical presentation and investigations, we finally reached the diagnosis of preeclampsia along with trichotillomania and dermatitis artefacta.

Considering her pregnancy and psychiatric illness, her management was carefully tailored. Behavioural therapy instituted in the form of habit reversal, relaxation training sessions, and competing response training. An occlusive dressing on her limbs was applied, following which her lesions began to heal rapidly. This acted as a negative feedback and also reconfirmed our diagnosis. Repeated counselling of the patient and family members was done to ensure adherence to therapy and the role of family support in mitigating the disease. Antipsychotic drugs were avoided and planned to be started after delivery, if needed. On further follow-up of the patient, a significant improvement was noticed in her behaviour with almost complete resolution of her skin lesions.

## 3. Discussion

Psychocutaneous disorders encompass a rare group of dermatological conditions as a somatic manifestation of an underlying psychiatric illness and not fully explained by a known dermatological disease. This clinical entity provides a suitable explanation for numerous skin manifestations that had been unexplained till now. Advent of psychoneuroimmunology deciphers the intricate relationship between nervous and immune system, mediated through the neuropeptides as shown in the work by Panconesi and Hautmann [1] affecting the lymphoid organs through sympathetic and peptidergic nerve fibres. Direct effect of neuropeptides upon lymphocytes, macrophages, and cells on skin (Langerhans cells and fibroblasts) has also been studied [1]. In light of this, various skin manifestations in stressed and anxious individuals could be attributed to decreased immunity. According to the Diagnostic and Statistical Manual (DSM) of Mental Disorders, fourth edition, as proposed by the American Psychiatric Association [2], dermatitis artefacta is a cutaneous manifestation of an underlying factitious disorder and trichotillomania is primarily a variety of impulse control skin diseases.

Factitious dermatitis or dermatitis artefacta is a self-inflicted dermatoses showing preponderance in females (male-to-female ratio varying from 20 : 1 to 4 : 1) as in the study by Koblenzer and Rogers et al. [3, 4]. There is intentional production and continuous feigning of illness and denial of the self-inflicted nature of dermatoses, achieving a “sick role” being the underlying motive as shown by Phillips KA [5]. No external incentive, economic or legal, is apparent; rather, psychosocial stress in the form of childhood abuse, marital trauma, physical body changes, substance abuse, self-guilt, disturbed parent-child relationship, or depression [3] might be associated. According to Lyell, patients characteristically give a hollow history [6] and dissociation is evident from their self-inflicted skin lesions. Classical skin lesions are as described in our patient. Method of infliction can be scratching as seen in our patient or cutting, biting, and application of thermal burns or chemical substances.

Trichotillomania (a term coined by Hallopeau in 1889) is a body-focussed repetitive behaviour (BFRB) characterised by impulsive hair pulling followed by a sense of relief after the act. Hair pulling has been shown in 1.5% of males and 3.4% of females among college students, but the DSM-IV criteria were satisfied by only 0.5% according to Christenson et al. [7]. Oranje et al. studied that children and adolescents far outnumber the cases in adults [8]. According to Deaver et al., in children, it is a self-limited habit disorder [9], whereas, in adolescents and adults, an underlying anxiety disorder is usually present. Associated trichophagia and gastrointestinal bezoars might be associated.

Management of psychocutaneous disorders is primarily directed towards the underlying psychiatric illness, with conservative management of cutaneous lesions. A sympathetic and receptive approach is necessary for the detection of underlying emotional situation and precipitating environmental influences. A tailored psychotherapeutic approach involving cognitive behavioural therapy has been shown to be more effective than pharmacotherapy using tricyclic antidepressants or selective serotonin reuptake inhibitors (SSRIs).

## 4. Conclusion

Factitious dermatitis and trichotillomania are extremely rare psychocutaneous disorders, and their coexistence is not known to the best of our knowledge. The difference in the underlying psychoetiology might be responsible for the same. Borderline personality disorder commonly seen in trichotillomania may be rarely associated even with factitious dermatitis that could add a compulsive component in dermatitis artefacta. This could account for a possible explanation of their coexistence in our patient.
